# Photoabsorption and Photoionization of Acetaldehyde in the 10.8–21.4 eV Range

**DOI:** 10.1021/acsomega.5c08603

**Published:** 2025-11-12

**Authors:** Milton M. Fujimoto, Bruno Credidio, Manoel G. P. Homem, Ricardo R. T. Marinho, Gabriel L. C. de Souza, Frederico V. Prudente

**Affiliations:** † Departamento de Física, Universidade Federal do Paraná, 81531-980 Curitiba, Paraná, Brazil; ‡ Instituto de Física, Universidade Federal da Bahia, 40170-115 Salvador, Bahia, Brazil; § Departamento de Química, Universidade Federal de São Carlos, 13565-905 São Carlos, São Paulo, Brazil; ∥ Instituto de Física, 28127 Universidade de Brasilia, 70910-900 Brasília, Distrito Federal, Brazil; ⊥ Centro de Ciências da Natureza, Universidade Federal de São Carlos, 18290-000 Buri, São Paulo, Brazil

## Abstract

An experimental and theoretical investigation of acetaldehyde’s photoabsorption and photoionization in the vacuum-ultraviolet energy range is presented. The absorption cross sections and the ionization efficiency were measured using a double-ion-chamber spectrometer in the 10.8–21.4 eV and 13.5–21.4 eV ranges, respectively. No previous reports of these quantities were found in the literature in this energy range. Photoionization and neutral-decay cross sections were derived from these results. Theoretical calculations were performed using the Padé approximant technique and the single-center partial-wave expansion method, which were applied to solve the Lippmann–Schwinger equation to obtain the asymmetry parameters and the photoionization cross sections for the seven outermost valence orbitals. The calculations were performed in the energy range from near-threshold to 35 eV at static-exchange and static-exchange-polarization levels of approximation. Our results are contextualized with recent studies on dissociative photoionization channels and high-resolution photoelectron spectra, enabling correlations with ionic fragmentation pathways. Our experimental and theoretical results were compared between each other and with previous results from the literature, exhibiting good agreement with the available data and providing a comprehensive data set essential for modeling photochemical processes in atmospheric and astrophysical environments.

## Graphical abstract

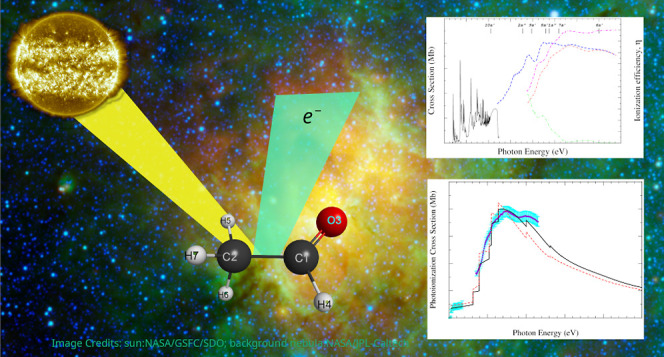

## 1 Introduction

Carbonyl compounds are among the most abundant organic compounds in the interstellar medium.^1–4^ Notably, one of the smallest aldehydes, acetaldehyde or ethanal (CH_3_CHO), was one of the first polyatomic molecules detected in outer space^2,5^ and later detected in the Hale-Bopp comet.^6^ Acetaldehyde also plays an important role in Earth’s atmosphere as a source of free radicals through photolysis processes.^7,8^ In this sense, detailed knowledge of photon interactions with these molecules over a wide energy range is important to better describe several reaction mechanisms and to achieve a deeper understanding of photochemistry in such environments.

The photon interaction with acetaldehyde has been investigated by various experimental techniques, for instance, by photoelectron^9,10^ and threshold photoelectron spectroscopy,^11^ photoelectron-photoion coincidence,^12^ ultraviolet photoabsorption spectroscopy^9,13–15^ and also by electron energy loss spectroscopy.^16–19^ Electron scattering studies can also be found; see, e.g., ref 20 and references therein. To our knowledge, absolute measurements of the photoabsorption spectrum were performed only by Limão-Vieira et al.,^7^ who reported cross-section results in the energy range from 3.0 to 11 eV. Theoretical studies are more scarce. In the vacuum-ultraviolet (VUV) energy range, only the work of Vega et al.^8^ that investigated the oscillator strengths for electronic transitions involving Rydberg states and cross sections for the dipole allowed photoionization channels of acetaldehyde is found.

The investigation of acetaldehyde has recently seen a revival. Dissociative photoionization channels were examined in detail by Recio et al. over the 10.2–19.5 eV range,^21^ while Wu et al. reported high-resolution photo- and threshold electron spectra relevant to the lowest-energy region.^22^ Earlier, Cvitaš, Güsten, and Klasinc^23^ had already provided high-resolution photoelectron spectra of acetaldehyde and its deuterated derivatives, assigning six vibrational modes of the cation and highlighting the strong influence of isotopic substitution (H → D) on the vibronic structure of the first ionization system. These pioneering results emphasized the role of oxygen lone-pair electrons in determining the cation geometry and established fundamental insights into the interplay between electronic and nuclear structure, though limited to the lowest ionization region. However, a unified set of absolute cross-section data (σ_a_ and σ_i_) spanning the entire energy region where these fragmentation and spectral features occur is still lacking in the literature.

The present work extends this body of knowledge by reporting the first absolute experimental measurements of the photoabsorption cross section (σ_*a*_) and ionization efficiency (η) of acetaldehyde in the vacuum-ultraviolet range from 10.8 to 21.4 eV. These values are essential for modeling acetaldehyde in astrophysical and atmospheric environments. On the theoretical side, we calculate photoionization cross sections and asymmetry parameters from threshold to 35 eV, employing the static-exchange and static-exchange-polarization approximations together with the Padé-approximant method based on a single-center partial-wave expansion of the Lippmann–Schwinger scattering equation.^24,25^ The resulting data set provides a unified description of the continuum dynamics for this molecule, complementing and extending recent experimental studies.

The organization of this paper is as follows: Section 2 describes the experimental procedure, Section 3 summarizes the theoretical and computational details, and Section 4 presents the calculated and experimental results together with comparisons to previous work. Concluding remarks are given in Section 5.

## 2 Experimental Section

The VUV absolute photoabsorption cross sections (σ_*a*_) and the ionization efficiency (η) of acetaldehyde were recorded at the Toroidal Grating Monochromator (D05A-TGM) beamline of the Brazilian Synchrotron Light Laboratory (LNLS) facility^26^ using a double-ion-chamber spectrometer,^27^ in the 10.8–21.4 eV photon-energy range (λ = 115–57 nm). A neon gas filter was responsible for minimizing contamination from higher-order harmonics, providing high-spectral purity radiation (around one ppm) up to absorption edge of neon (21.6 eV).,^28,29^ The absolute energy scale was calibrated, and the energy resolution (≈50 meV, corresponding to Δλ ≈ 0.14–0.53 nm across the studied energy range) was determined by observing the energy cutoff at this absorption edge. The photoabsorption experimental setup, procedures, and the evaluation of experimental uncertainties have been described in detail previously.^30–35^ Essentially, the results were obtained from the measurements of two ion currents (*i*_1_ and *i*_2_) collected by two successive plates of the same length (*l* = 66.5 ± 0.1 mm) positioned inside the absorption cell and separated by a small gap (0.5 mm). From these measurements, σ_*a*_ and η are given by^27^

1 $$\sigma_{a}\left(E\right)=\frac{1}{nl}\ln\left(\frac{i_{1}}{i_{2}}\right)$$

and

2 $$\eta \left(E\right)=\frac{{i_{1}}^{2}}{eI_{0}\left(i_{1}-i_{2}\right)}$$

respectively, where *n* is the molecular number density, *e* is the elementary charge, and *I*_0_ is the absolute photon intensity. The *I*_0_ was determined through measurements with Xe above its ionization energy (≈13.5 eV). The measured ionization efficiency (η) is a fundamental quantity derived from the experimental setup, defined as the ratio of the photoionization cross section (σ_i_) to the total photoabsorption cross section (σ_a_)­[Fn fn1]

3 $$\eta =\frac{\sigma_{i}}{\sigma_{a}}$$

This quantity directly represents the probability that an absorbed photon leads to ionization rather than a neutral decay process. While related to the Ion Yield (the ratio of ions produced to incident photons), the Ionization Efficiency is the key experimental result for modeling photoionization dynamics. Since our experiment yields both σ_a_ and η, the absolute photoionization cross section is calculated as σ_i_ = η × σ_a_. Therefore, our η results are reported starting from 13.5 eV. Details of the procedures were given elsewhere; see, e.g., ref 34. The acetaldehyde sample pressure in the absorption cell, kept in the range of 40–100 mTorr (5.3 to 13.3 Pa), was monitored by an absolute capacitance manometer (MKS Baratron model 624B01T), and the ion currents were measured by two electrometers (Keithley model 6514). The procedure enables us to determine the accuracy of the photoabsorption cross section and the ionization efficiency to within ±5% and ±10%, respectively. From these results, photoionization (σ_*i*_ = ησ_a_) and neutral-decay [σ_n_ = (1 – η)­σ_a_] cross sections were derived with an accuracy within ±11%.

The acetaldehyde sample was purchased from Sigma-Aldrich with a stated purity higher than 99%. We have used the freeze–pump–thaw purification method, with the vial connected to the experimental chamber through a needle valve. The purity of the sample was verified using a quadrupole mass spectrometer (Balzers Prisma QMA200) to check for contaminants, and none, including water, were considerably detected.

## 3 Theory and Computational Details

To compute the theoretical cross sections and asymmetry parameters presented here, we use ePolyScat.E3,^24,25^ which employs the Schwinger variational method combined with Padé Approximants to generate continuum wave functions and compute dipole matrix elements corresponding to direct photoionization of molecules induced by single-photon absorption. As the method has been described in detail in several previous publications,^24,25,36^ we provide here only a simplified overview of the calculations.

Observable quantities in photoionization processes, such as cross sections and angular distributions, are obtained by evaluating the dipole matrix elements, which quantify the transition amplitudes between bound and continuum states induced by interaction with electromagnetic radiation. The electric dipole operator in these matrix elements can typically be expressed in two equivalent forms. In the length form

4 $$I_{k,\mathrm{n̂}}^{\left(L\right)}=k^{1/2}\left\langle \psi_{i}\left|r.\mathrm{n̂}\right|\psi_{k}^{\left(-\right)}\right\rangle$$

and also in the velocity form

5 $$I_{k,\mathrm{n̂}}^{\left(V\right)}=\frac{k^{1/2}}{E}\left\langle \psi_{i}\left|\nabla .\mathrm{n̂}\right|\psi_{k}^{\left(-\right)}\right\rangle$$

Here, ψ_*i*_ denotes the initial bound state, representing the ground-state wave function of the neutral molecule, while ψ_**k**_^(−)^ corresponds to an antisymmetrized product of the molecular ion wave function and the outgoing photoelectron. The vector **k** represents the momentum of the continuum electron, and the superscript ^(−)^ indicates that ψ_**k**_^(−)^ fulfills the boundary condition for incoming spherical waves. The parameter *E* refers to the photon energy, and *n̂* defines the orientation of the linearly polarized light field. The evaluation of these dipole matrix elements imposes selection rules that govern dipole-allowed transitions.

Although the length and velocity forms of the dipole matrix elements are theoretically equivalent, numerical implementations may lead to discrepancies depending on the quality of the continuum and bound wave functions. To mitigate such inconsistencies, ePolyScat also employs a “mixed” approximation (*M*), in which the dipole operator is expressed in the length form regarding the bound state and in the velocity form with respect to the continuum state. This approach reduces sensitivity to numerical inaccuracies and improves the reliability of the computed matrix elements. The use of a mixed gauge in describing molecular photoionization processes is a well-established approach in the literature, with several studies discussing its advantages.^37,38^

For a linearly polarized photon beam, the differential cross section for the photoelectron angular distribution (PAD) averaged over molecular orientations is given by^24,25,39^

6 $$\frac{d\sigma_{i}^{\left(L,V\right)}}{d\Omega_{k}}=\frac{\sigma_{i}^{\left(L,V\right)}}{4\pi }\left[1+\beta_{k}^{\left(L,V\right)}P_{2}\left(\cos\theta \right)\right]$$

where σ_*i*_^(*L*,*V*)^ is the photoionization cross section and β_**k**_^(*L*,*V*)^ is the asymmetry parameter, obtained in the length (*L*) or velocity (*V*) form of the dipole operator. The expressions relating σ_*i*_^(*L*,*V*)^ and β_**k**_^(*L*,*V*)^ to the integral dipole matrix elements are provided in previous references, such as ref 36. *P*_2_(cos θ) is the Legendre polynomial of the second order, where θ is the angle of the ejected electron momentum vector **k** with respect to the photon polarization vector. For comparison with gas-phase experimental measurements, the laboratory-frame photoionization cross section (PICS) is obtained by averaging over all molecular orientations relative to the light polarization vector, *n̂*, and integrating over all photoelectron emission directions, *k̂*.

The neutral and ion molecular wave functions used in eqs 4 and 5 are obtained using the Gaussian 03 ^40^ employing a near-Hartree–Fock self-consistent field (HF-SCF) approach with the aug-cc-pVTZ^41^ basis set. The molecular ion wave function is constructed using the frozen-core approximation,^42^ in which the spin–orbitals of the neutral ground state are retained, except for a vacancy introduced in the orbital corresponding to the ionized electron. The experimental molecular geometry of nuclei of acetaldehyde^43^ was used, and the molecule represented by *C*_s_ point group providing a total energy of −152.97925 au and an electric dipole moment of 1.214 D. The ground-state electronic configuration of the neutral molecule, within this level of approximation, is given by

$${\left(1a^{\prime }\right)}^{2}{\left(2a^{\prime }\right)}^{2}{\left(3a^{\prime }\right)}^{2}{\left(4a^{\prime }\right)}^{2}{\left(5a^{\prime }\right)}^{2}{\left(6a^{\prime }\right)}^{2}{\left(7a^{\prime }\right)}^{2}{\left(8a^{\prime }\right)}^{2}{\left(1a^{\prime \prime }\right)}^{2}{\left(9a^{\prime }\right)}^{2}{\left(2a^{\prime \prime }\right)}^{2}{\left(10a^{\prime }\right)}^{2}$$

In principle, ionization calculations were performed for all nine valence orbitals; however, detailed results are presented only for the seven outermost orbitals, which contribute most significantly to the photoionization process.

The calculations for photoelectron wave function, dipole matrix elements, and observable of interest were performed using the e-PolyScat-E package that employs the [*N*/*N*] Padé’s approximant technique along with nuclei-fixed approximation and the single-center partial-wave expansion method to solve the Lippmann–Schwinger equation.^24,25^

At the static-exchange (SE) level of approximation, the SE potential was fully derived from a near-Hartree–Fock self-consistent-feld (HF-SCF) wave function. In contrast, at the static-exchange-polarization (SEP) level, the correlation-polarization potential was obtained from a parameter-free local density model described by Perdew and Zunger^44^ and added to the interaction potential. To generate the asymptotic part of the correlation-polarization potential, the average static dipole polarizability α_0_ = 30.975 au was used.

In addition to molecular orbital descriptions, the ePolyscat program requires specific molecular properties of acetaldehyde, such as orbital ionization energies and molecular polarizability. In these calculations, we use experimental ionization energy values from Yencha et al.^11^ for the six outermost valence orbitals: 10a′, 2a″, 9a′, 1a′′, 8a′, and 7a′. We have also calculated ionization energies for all nine valence orbitals and used the theoretical values for the three innermost orbitals: 6a′, 5a′, and 4a′. These values are presented in Table 1. The (vertical) ionization energies were calculated with the CFOUR program^45,46^ through the use of the equation-of-motion ionization potential coupled-cluster with single and double excitations approach (EOMIP-CCSD)^47^ with the aug-cc-pVTZ^41^ basis set. Regarding the calculated vertical ionization energies presented in Table 1, our EOMIP-CCSD values are in good agreement with the experimental data. We have also included a comparison with the recently published theoretical vertical ionization energies by Recio et al.^21^ The overall excellent consistency between our theoretical results, the reference experimental data, and the values reported by Recio et al. further validates the quality of the initial molecular orbital descriptions used in our scattering calculations.

**Table 1:** Vertical Ionization Energies (VIEs) for the Nine Valence Orbitals of Acetaldehyde (in eV)^a^

orbital	Exp. (Yencha et al.^11^) (VIE/eV)	Recio et al. (2024)^21^ (VIE/eV)	this work (EOMIP-CCSD/eV)
10*a*′	10.228	10.21	10.227
2*a*″	13.093	13.31	13.582
9*a*′	13.93	14.53	14.536
1*a*″	15.5	N/A	15.920
8*a*′	15.20	15.69	15.543
7*a*′	16.37	N/A	16.829
6*a*′	19.4	N/A	20.013
5*a*′	N/A	N/A	23.111
4*a*′	N/A	N/A	24.597

a The experimental values of Yencha et al. were taken from ref 11. For comparison, the recent theoretical VIEs of Recio et al. (using the CASPT2 method) are also included from ref 21. The theoretical values reported in this work were calculated at the EOMIP-CCSD/aug-cc-pVTZ level. (N/A = Not available).

As the ePolyScat computational program employs a partial wave expansion to represent the scattering wave function, enabling an accurate treatment of electron–molecule interactions, it is essential to verify the convergence parameters to ensure the reliability of the results. The wave functions, interaction potentials, and the related matrices were all single-center expanded around the center-of-mass of the molecule in terms of the symmetry-adapted functions.^48^ Our partial wave expansion for the molecular orbitals and the scattering wave functions included up to *l*_max_ = 30. These expansions give orbital normalization integrals better than 0.99 for all molecular orbitals except the innermost, which has 0.97. The *l*_maxK_ = 15 for *K*-matrix, which is used to obtain the photoelectron wave function. These cutoff parameters were also used for the continuum orbitals, and all calculated results were converged within 12 iterations, with an accuracy better than 3%.

## 4 Results and Discussion

Our experimental η, σ_*a*_, σ_*i*_, and σ_*n*_ data in the 10.8–21.4 eV range are presented in Figure 1. The complete set of experimental cross section values (σ_*a*_, η, σ_*i*_, and σ_*n*_) is listed in Table S1 of the Supporting Information. The primary purpose of Table S1 is to provide the full data set, which is necessary for the calculation of integral quantities like the static dipole polarizability (discussed later). The existing σ_*a*_ experimental data of Limão-Vieira et al.^7^ in the energy range from 6 to 10.8 eV are also shown. Although there is a small gap in photon energy between our absorption cross section and that of Limão-Vieira et al., the two data sets exhibit a smooth and continuous transition, suggesting overall consistency.

**Figure 1:**
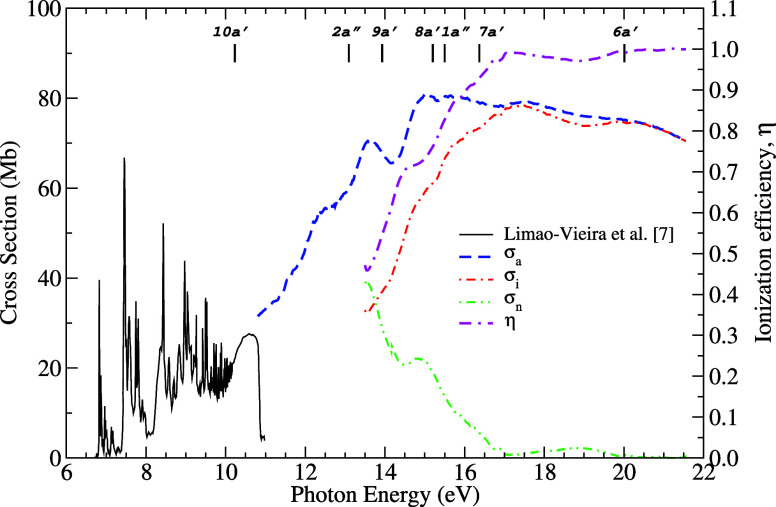
Present experimental results for acetaldehyde: (--·-), ionization efficiency (η); (---), photoabsorption (σ_*a*_); (-·-·-), photoionization (σ_*i*_); (-··-), neutral-decay cross section (σ_*n*_). Below 10.8 eV are also shown the (—) photoabsorption data of Limão-Vieira et al. obtained from ref 7. The vertical bars represent the ionization energy of a corresponding orbital.

To extend the energy range of the dipole oscillator strength distribution (d*f*/d*E* = 9.112 × 10^–3^σ_*a*_),^49^ we combined the present σ_*a*_ data with previously published results,^7^ which cover photon energies up to 10.6 eV. This allowed us to determine the static dipole polarizability of acetaldehyde by applying the S(−2) sum rule. See Figure S1 of Supporting Information for details. The result of (32.2 ± 0.5) a.u. reasonably agrees with the experimental value of 31.0 au obtained from refractive index measurements.^50^ The value of polarizability considered in our SEP level calculation is 30.975 au, which is obtained from the B3LYP/aug-cc-pVTZ level using Gaussian 03.

In Figure 1, our experimental σ_*i*_ is obtained from η and σ_*a*_. The η values approach unity above 20 eV, indicating that photon absorption in this energy range primarily leads to ionization. This behavior is also reflected in the convergence of the absorption cross section (σ_*a*_) to the ionization cross section (σ_*i*_) near 20 eV. Conversely, the neutral cross section (σ_*n*_), which represents photon absorption processes that do not lead to ionization, decreases with increasing energy and effectively vanishes above 20 eV.

In Figures 2 and 3, the calculated partial photoionization cross sections (PICS) for each dipole-allowed ionization channel (ϕa′ → *ka*′, *ka*″ and ϕa″ → *ka*′*ka*″) from seven valence orbitals, are shown in three levels of approximations: SE, SEP, including or not polarization effects and *L* dipole forms. Figure 2 shows our partial PICS for (10*a*′)^−1^, ${\left(2a^{\prime \prime }\right)}^{-1}$ , (9*a*′)^−1^ ionic states, and ${\left(1a^{\prime \prime }\right)}^{-1}$ . Figure 3 show (8*a*′)^−1^, (7*a*′)^−1^, and (6*a*′)^−1^ ionic states. We are not comparing these results with those of other dipole forms (*M* and *V*), although they have slightly different magnitudes, the qualitative behavior is the same.

**Figure 2:**
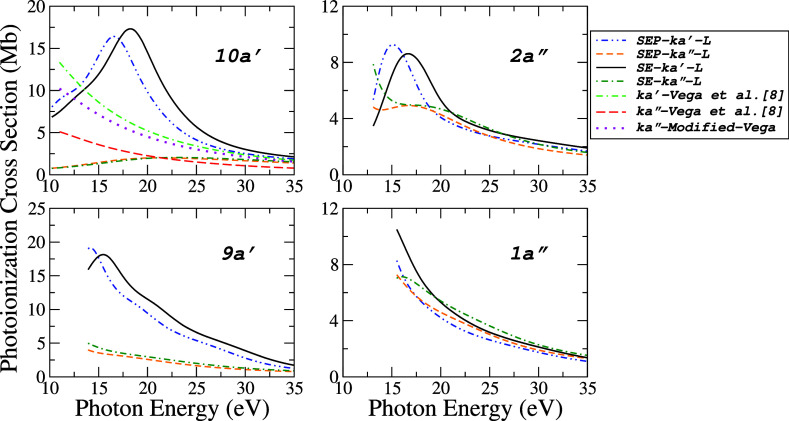
Contributions to the partial photoionization cross sections for the four outermost valence orbitals (10*a*′, 2*a*″, 9*a*′ and 1*a*″) of acetaldehyde obtained in *L* form: (-··-), SEP-*ka*′; (---), SEP-*ka*″; (—), SE-*ka*′; (-·-), SE-*ka*″. Literature: for the 10*a*′ orbital, the data of Vega et al. obtained from ref 8 are also shown: (--·-), *ka*″ Vega et al.^8^ (*ks* + *kd*_*xy*_ + *kd*_*x* ^2^+*y* ^2^_ + *kd*_*z* ^2^_); (−−), *ka*″(*kd*_*yz*_) and (······) *ka*″-modified-Vega: *ka*″(*kd*_*yz*_) plus *kd*_*xz*_ by Vega et al.^8^

**Figure 3:**
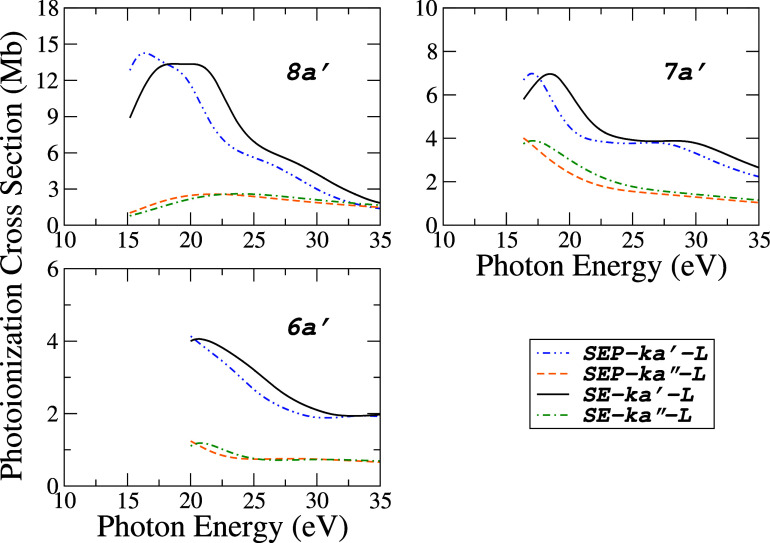
Contributions to the partial photoionization cross sections for the three inner valence orbitals (8*a*′, 7*a*′ and 6*a*′) of acetaldehyde obtained in *L* form: (-··-), SEP-ka′; (---), SEP-ka″; (—), SE-ka′; (-·-) and SE-ka″.

The partial PICS for *a*′ → *ka*′, *ka*″ for (10*a*′)^−1^ molecular orbital calculated by Vega et al.^8^ are also compared. When the partial PICS exhibits a resonant feature, such as the peaks observed around 16–18 eV in the (10*a*′)^−1^ channel, the corresponding peaks at the SEP level are generally shifted by approximately 1.6–1.7 eV to lower energies compared to those at the SE level. This energy shift of the spectral feature likely results from the stabilization of the ion–photoelectron complex, caused by the distortion of the electronic cloud of the ion in response to the electric field generated by the ejected electron. In contrast to our results for (10*a*′)^−1^ configuration, the calculations by Vega et al. do not show a corresponding peak for the *ka*′ transition in this energy range. This suggests that, in their model, the maximum of the cross section may lie below the ionization threshold, implying stronger polarization effects than those considered in our SEP approach.

Another difference is observed at the *ka*″ channel, while our results tend to zero near the threshold, theirs are increasing. As the origin of this discrepancy remains unclear, we attribute it to differences in the computational methodologies. While their approach is well-suited for describing autoionization processes involving excitations to Rydberg states, our method is primarily focused on direct ionization. Their results are qualitatively more similar to our results for the (9*a*′)^−1^ state. One point we would like to discuss about Vega et al.^8^ work is that their results for *ka*′ were obtained from contributions of (*ks* + *kd*_*xy*_ + *kd*_*x* ^2^+*y* ^2^_ + *kd*_*z* ^2^_) which we would expect to be equivalent to our calculations, although their calculation considers Rydberg “atomic” orbitals while ours were obtained considering molecular orbitals. For the *ka*″ channel, they commented that they considered only *kd*_*yz*_’s contribution. However, we understand that for this channel, the *kd*_*xz*_ channel should also be taken into account. Since the only difference between these two orbitals lies in their spatial orientation around the bond axis, we believe that the cross section reported by Vega et al. for the *ka*″ orbital should be twice as large as the value they published. In Figure 2, we present both sets of results for the *ka*″ orbital: the original values reported by Vega et al. and a “modified” version, which corresponds to twice their reported values. The “modified” *ka*″ data set shows better agreement with our corresponding results at higher energies than the original data reported by Vega et al.^8^ To the best of our knowledge, no literature data is available for the other valence orbitals for comparison.

In Figures 4 and 5, the calculated PICS for seven valence orbitals are presented using six levels of approximation: SE, SEP (each evaluated without and with polarization effects), and employing the *L*, *M*, and *V* dipole forms. The resulting curves represent the sum of *ka*′ and *ka*″ continuum symmetries for a given orbital. Figure 4 presents the results for four orbitals: (10*a*′)^−1^, ${\left(2a^{\prime \prime }\right)}^{-1}$ , (9*a*′)^−1^ and ${\left(1a^{\prime \prime }\right)}^{-1}$ ionic states, while Figure 5 shows the results for the (8*a*′)^−1^, (7*a*′)^−1^, and (6*a*′)^−1^ states. The results of Vega et al.^8^ from Figure 2 are summed and compared in Figure 4, along with the corresponding comparison using the “modified-Vega” data set for (10*a*′)^−1^ state.

**Figure 4:**
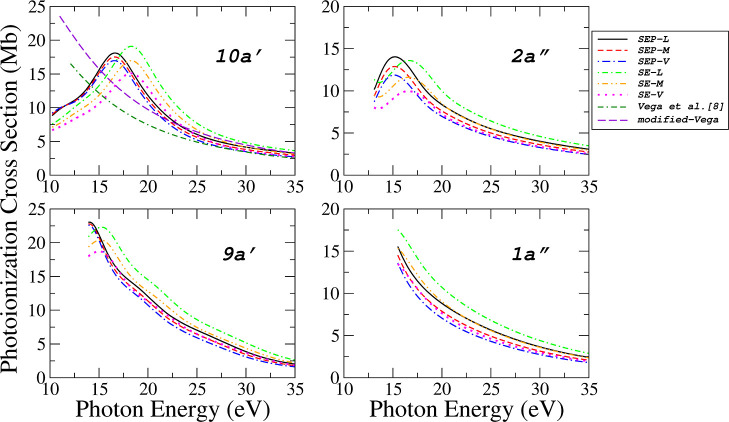
Photoionization cross sections for the four outermost valence orbitals (10*a*′, 2*a*″, 9*a*′ and 1*a*″) of acetaldehyde obtained in six levels of calculations: (—), SEP-*L*; (---), SEP-*M*; (−·−), SEP-*V*; (-·-), SE-*L*; (-··-), SE-*M*; (······), SE-*V*; (--·-) results of Vega et al. obtained from ref 8 and (−−) modified-Vega (see the text).

**Figure 5:**
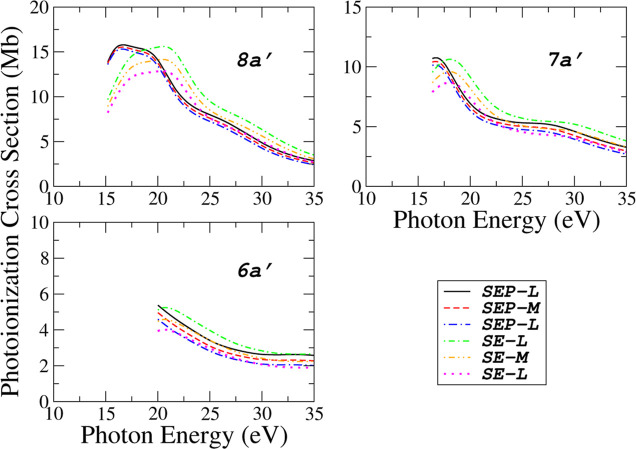
Photoionization cross sections for the three inner valence orbitals (8*a*′, 7*a*′ and 6*a*′) of acetaldehyde obtained in six levels of calculations: (—), SEP-*L*; (---), SEP-*M*; (−·−), SEP-*V*; (-·-), SE-*L*; (-··-), SE-*M* and (······), SE-*V*.

Above 21 eV, our results present a good agreement with Vega et al. and “modified-Vega”; below that, we have a qualitative disagreement. In the absence of other results for comparison, our calculations using different levels of approximation provide a range of values within which the experimental results are expected to lie. For this molecule, at a given level of polarization, the *L* approximation yields the highest cross section values, whereas the *V* approximation provides the lower bound. If the exact wave functions for the molecule and the photoelectrons were used, one would expect these values (*L* and *V*) to be equivalent. In practical applications, the *L* approximation exhibits increasing deviation from the *V* approximation as the energy decreases. To circumvent this limitation, the ePolyScat has implemented an intermediate approach known as the “mixed” approximation (denoted here as *M*). This method effectively combines the length and velocity forms of the dipole operator to improve numerical stability without requiring exact wave functions. As a result, the outcomes produced by this *M* approximation generally fall between those of the *L* and *V* approximations across nearly the entire photon energy range, as illustrated in Figures 4 and 5.

Our theoretical photoionization cross sections summed for all nine valence orbitals of acetaldehyde leading to the (10*a*′)^−1^, ${\left(2a^{\prime \prime }\right)}^{-1}$ , (9*a*′)^−1^, ${\left(1a^{\prime \prime }\right)}^{-1}$ , (8*a*′)^−1^, (7*a*′)^−1^, (6*a*′)^−1^, (5*a*′)^−1^ and (4*a*′)^−1^ ionic states are shown in Figure 6, at the same six levels of calculations reported previously in Figures 4 and 5. Although we have calculated the contributions from all nine valence orbitals of acetaldehyde, only the seven outermost orbitals contribute significantly to the summed PICS.

**Figure 6:**
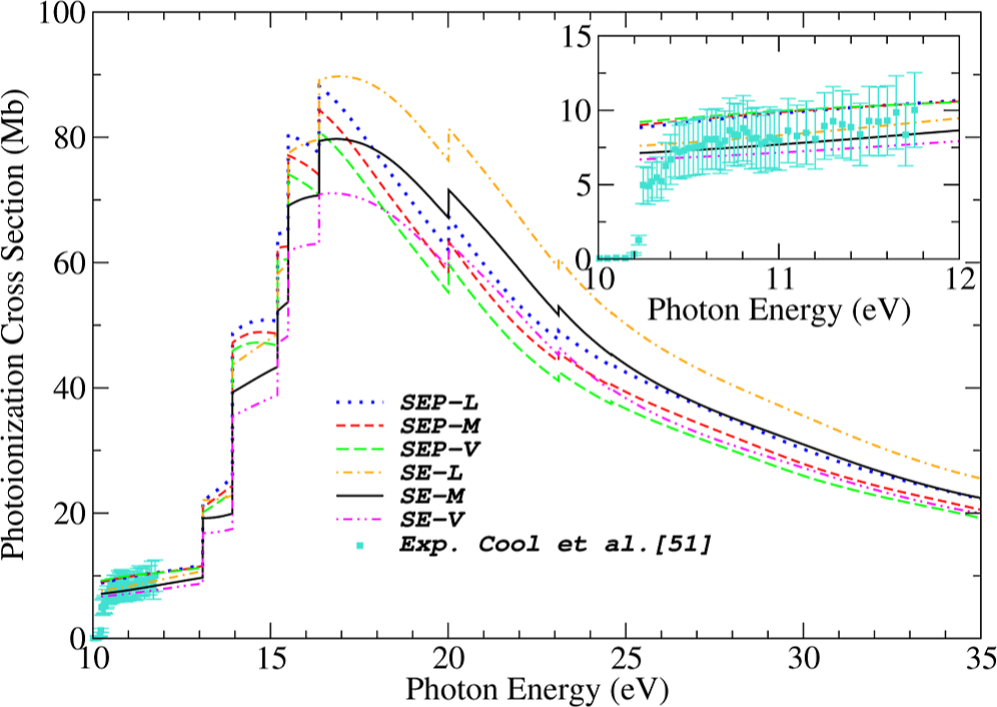
Theoretical photoionization cross sections sum of the nine (9) valence orbitals for acetaldehyde obtained at the SEP level: with (······) dipole-length (*L*), (---) dipole-mixed (*L*) and (−−) dipole-velocity (*L*)­forms; at the SE level: with (·-·-) dipole-length (*L*), (—) dipole-mixed (*M*) and (-··-) dipole-velocity (*V*) forms. Experimental data of Cool et al. obtained from ref 51 (■). Error bars indicate a 25% uncertainty.

As shown in Figure 6, the contributions of the two innermost valence orbitals (5*a*′ and 4*a*′) have a negligible effect on the overall sum. Therefore, detailed results for the (5*a*′)^−1^ and (4*a*′)^−1^ orbitals are not presented in this work. The sharp increase in the cross section curve suggests the onset of a new ionization channel. We do not observe major differences in the cross section profile comparing the SEP and SE calculations, except near the maximum around 17 eV. It is observed that the largest cross section magnitude is obtained using the *L* approximation, while the *V* approximation yields the smallest value for this molecule. Near the ionization threshold, the only experimental data available to our knowledge are those reported by Cool et al.^51^ These measurements were obtained using flame-sampling photoionization mass spectrometry (PIMS) combined with synchrotron radiation. As shown in the inset of Figure 6, within the 10 to 12 eV energy range, our calculated photoionization cross sections, across all levels of theory, exhibit good agreement with the experimental data of Cool et al.

In Figure 7, we present our theoretical photoionization cross section, obtained by summing the contributions from all nine valence orbitals of acetaldehyde, and compare it with our present experimental measurements. The calculations are shown at two levels of approximation: SEP-*M*, SE-*M*. The mixed (*M*) approximation is used for comparison, as it provides better agreement in magnitude with the experimental results.

**Figure 7:**
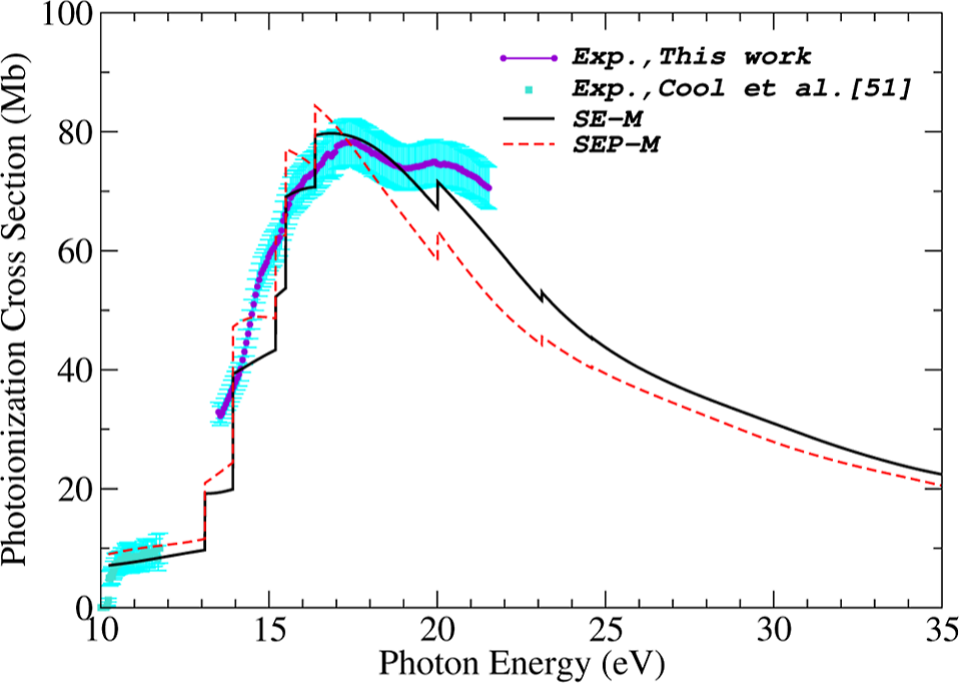
Theoretical summed photoionization cross sections sum of the nine (9) valence orbitals for acetaldehyde obtained in the dipole-mixed (*M*) form at (—) SE and (---) SEP level. Experimental data of Cool et al. obtained from ref 51 (■). Error bars indicate a 25% uncertainty; This work: (•—•). Error bars represent a 5% uncertainty.

In a previous study by our research group on pyrrole,^30^ we demonstrated that incorporating polarization effects is essential near the ionization threshold. However, better agreement with experimental data was achieved without polarization effects in the region around the cross section maximum, approximately 7 eV above the ionization threshold.

For acetaldehyde, the inclusion of polarization effects does not substantially influence the cross section near the ionization threshold, as no significant differences are observed between the SE and SEP results. Unfortunately, our experimental measurements start at 13.5 eV, which prevents direct comparison in this region. However, the agreement between the experimental data of Cool et al.^51^ and our SE-M and SEP-M results demonstrates the robustness of our computational methods. Near the cross-section maximum, around 17 eV, the SE results show better agreement with the experimental data compared to those obtained at the SEP level. This suggests that the potential model employed to account for polarization effects may be overestimating their contribution in the photoionization process. A partial explanation for this behavior is discussed in Medeiros et al.,^30^ where it is noted that, in photoionization, the Coulomb interaction plays a more dominant role than polarization effects, except when the photoelectron has very low kinetic energy.

Another point to be highlighted is that the experimental data below 15.3 eV are higher than our theoretical results. This discrepancy may arise from the fact that the experimental measurements include contributions from both direct ionization and autoionization, whereas the ePolyScat computational package calculates the direct photoionization cross section. Furthermore, it is worth noting that the experimental data exhibit a second peak in the cross section at approximately 20 eV. According to our theoretical results, this peak arises from the contribution of the 6*a*′ orbital, which has an ionization potential of 20.013 eV. Unfortunately, a direct comparison between our experimental results and the measurements reported by Cool et al.^51^ is not possible due to the lack of overlap in the energy ranges. Nevertheless, our theoretical results exhibit good agreement with both sets of experimental data.

Furthermore, by considering the ionized states of the parent cation, a correlation can be established with the ionic fragments reported by Recio et al.^21^ and Wu et al.,^22^ providing additional insight into the fragmentation pathways at higher ionization energies. Since the ionization energy of acetaldehyde leading to the cationic ground state (X̃ ^2^A′, arising from ionization of the 10a^′^ orbital) is 10.228 eV and the first excited cationic state (Ã ^2^A^″^, from ionization of the 2a^″^ orbital) lies at 13.093 eV, all fragments with appearance energies­(AEs) below 13.093 eV must originate from the dissociation of the parent ion in its ground electronic state. The parent cation is therefore produced in the X̃ ^2^A^′^ state, possibly with vibrational excitation, and the observed fragments—CH_3_CO^+^ (AE = 10.87,^21^ 10.89 ^22^ eV), HCO^+^ (AE = 11.84,^21^ 11.54 ^22^ eV), and CH_4_^+^ (AE = 12.74,^21^ 12.85 ^22^ eV)—are consistent with this picture, as their appearance energies lie below the threshold of the Ã ^2^A″ state. The CH_2_CO^+^ fragment, with an appearance energy of 13.10 eV, can also be generated from the ${\left(2a^{\prime \prime }\right)}^{-1}$ state (Ã ^2^A′).

In addition, the ionic states (9*a*′)^−1^ (C̃ ^2^A′, IE = 13.93 eV), and (8*a*′)^−1^ (D̃ ^2^A′, IE = 15.2 eV) play a role in the formation of higher-energy fragments. The fragments CH_3_^+^ (AE = 14.09 ^21^ eV) and CH_2_^+^ (AE = 15.1 ^21^ eV) may be produced via ionization to the (9*a*′)^−1^ state, since the ionization energy of the (8*a*′)^−1^ state is higher than their appearance energies. This suggests that dissociation pathways involving excited cationic states contribute to the observed fragmentation pattern at higher photon energies.

In Figures 8 and 9, the asymmetry parameters β as a function of photon energy for each molecular orbital are shown at four levels of calculation: SE-*L*/*V* and SEP-*L*/*V*. The Figure 8 shows the transitions leading to the ionic states (10*a*′)^−1^, ${\left(2a^{\prime \prime }\right)}^{-1}$ , (9*a*′)^−1^, ${\left(1a^{\prime \prime }\right)}^{-1}$ while Figure 9 the transitions to (8*a*′)^−1^, (7*a*′)^−1^ and (6*a*′)^−1^ states, respectively. These results are presented here for the first time, as no corresponding data have been found in the literature to date.

**Figure 8:**
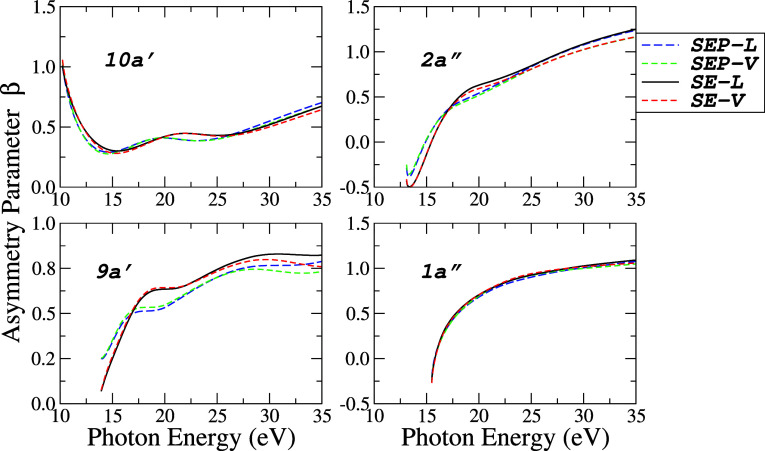
Photoelectron asymmetry parameters for the four (4) outermost valence orbitals (10*a*′, 2*a*″, 9*a*′, and 1*a*″) of acetaldehyde at four levels of approximations: (−−) SEP-*L*; (---) SEP-*V*; (—) SE-*L* and (−–−) SE-*V*.

**Figure 9:**
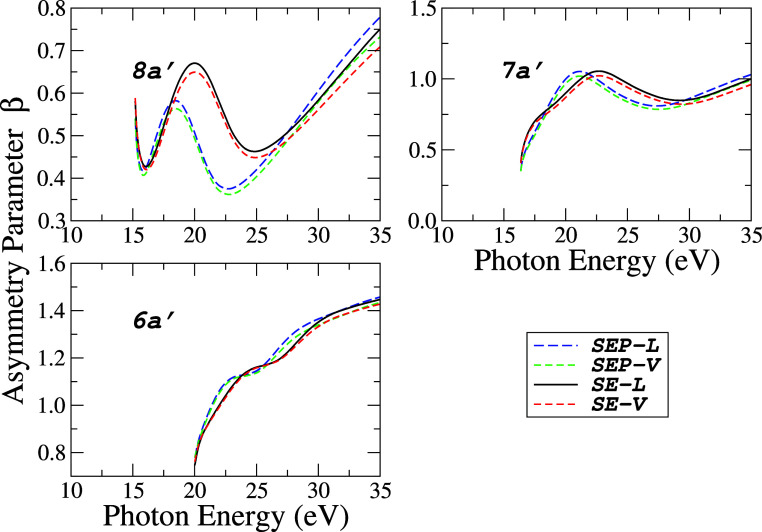
Photoelectron asymmetry parameters for the three (3) inner valence orbitals (8*a*′, 7*a*′, and 6*a*′) of acetaldehyde at four levels of approximations: (−−) SEP-*L*; (---) SEP-*V*; (—) SE-*L* and (- - -) SE-*V*.

In general, the asymmetry parameter values are much less sensitive to the level of approximation than the partial cross sections. The results for β with *L* and *V* are almost indistinguishable, and in the SEP approximation, they present their maxima and minima slightly shifted to lower energies compared to the SE approximation. The profile of the β curve as a function of energy can be used by experimentalists to assign the σ or π character of the molecular orbitals in the analysis of a photoelectron spectrum.^53^ In the case of acetaldehyde, the *a*′ orbitals exhibit σ character while *a*″ orbitals possess π character. The β curve associated with π orbitals typically exhibits a rapid increase from the ionization threshold, reaching a plateau region, such as observed for 2*a*″ and 1*a*″ (see Figure 8). In contrast, the asymmetry parameter for σ orbitals tends to display more pronounced oscillatory behavior and lacks a well-defined characteristic profile.

Another noteworthy aspect is that π orbitals exhibit a greater variation in the range of β values. Specifically, the difference between the maximum and minimum β values for a given orbital within the studied energy range for π orbitals lies approximately between 1.3 and 1.7. In contrast, for σ orbitals, this variation is significantly smaller, ranging from about 0.3 to 0.7. Overall, these distinctions may be especially valuable for experimental determinations of orbital symmetry in complex molecular systems.

## 5 Conclusions

In this work, we have conducted a combined experimental and theoretical investigation of the photoabsorption and photoionization processes of acetaldehyde (CH_3_CHO) in the vacuum-ultraviolet (VUV) energy range. Absolute photoabsorption cross sections and ionization efficiencies were measured between 10.8–21.4 eV and 13.5–21.4 eV, respectively. These results allowed us to derive accurate photoionization and neutral-decay cross sections. On the theoretical side, we employed the ePolyScat package to compute photoionization cross sections and asymmetry parameters for nine valence orbitals, using the static-exchange-polarization approximation and Padé approximants. Among these, only seven orbitals contribute significantly to the photoionization process. These calculations include, for the first time, a detailed characterization of photoelectron angular distributions for acetaldehyde.

The theoretical and experimental results show good agreement with each other and with previous limited data available in the literature. Our findings highlight the importance of polarization effects and validate the use of the mixed dipole approximation for cross-section predictions. They also offer new reference data for modeling the photophysics of small carbonyl molecules in interstellar and atmospheric environments. Furthermore, by combining our absolute photoionization cross section with recent ionic fragmentation branching ratios (e.g., from PEPICO studies^22^), a complete set of absolute cross sections for the formation of ionic fragments can now be derived, which is essential for detailed photochemical models of the interstellar medium and planetary atmospheres. To our knowledge, no previous study has reported the photoabsorption, photoionization, and neutral-decay cross sections of acetaldehyde over this photon energy range. The same applies to the photoelectron asymmetry parameters, which are presented here for the first time. We hope these findings will encourage future experimental and theoretical work on this and related molecular systems.

## Supplementary Material



## Footnotes

1 Currently, the TGM beamline is decommissioned.

## Notes

Supporting Information for auxiliary table containing numerical values of the experimental data for some selected photon energies and auxiliary figure containing (d*f*/d*E*)/*E* ^2^ in function of photon energy for acetaldehyde. This quantity was used to determine the static dipole polarizability of acetaldehyde by applying the S(−2) sum rule (PDF)

## #

Treunrenburgstraat 26, 5613 EB Eindhoven, The Netherlands
