# Supplementary material for: Photoabsorption and Photoionization of Acetaldehyde in the 10.8–21.4 eV Range
Source: ACS Omega. 2025 Nov 12;10(46):56500–10. doi: 10.1021/acsomega.5c08603 (PMC12658651; doi:10.1021/acsomega.5c08603)
Supplement: Supplementary file 1 [file ao5c08603_si_001.pdf]

Supplementary Material - Photoabsorption and Photoionization of Acetaldehyde in the 10.8–21.4 eV Range

Milton M. Fujimoto,[†] Bruno Credidio,[‡,⊥] Manoel G. P. Homem,[¶] Ricardo R. T. Marinho,[§,‡] Gabriel L. C. de Souza,[‖] and Frederico V. Prudente[*,‡]

†*Departamento de Física, Universidade Federal do Paraná, 81531-980, Curitiba, PR, Brasil*

‡*Instituto de Física, Universidade Federal da Bahia, 40170-115 Salvador, BA, Brasil*

¶*Departamento de Química, Universidade Federal de São Carlos, 13565-905 São Carlos, SP, Brasil*

§*Instituto de Física, Universidade de Brasilia, 70910-900, Brasília, DF, Brasil.*

‖*Centro de Ciências da Natureza, Universidade Federal de São Carlos, 18290-000, Buri, SP, Brazil.*

⊥*Current address: Treunrenburgstraat 26 5613 EB Eindhoven The Netherlands*

E-mail: prudente@ufba.br

Table S1. Experimental ionization quantum yields (η), photoabsorption (σ_a), photoionization (σ_i) and neutral-decay (σ_n) cross sections for acetaldehyde. Cross sections are given in units of Mb (10^{-18} cm^2). An overall uncertainty of 10% was determined in the η values, 4% in the σ_a, and 11% in the σ_i and σ_n.

Figure S1. $(df/dE)/E^2$ in function of photon energy for acetaldehyde. Below 0.4 a.u. (10.8 eV) the data were obtained using the σ_a of Limão-Vieira *et al.*[9]; above 0.8 a.u. (21.5 eV) the data were extrapolated using the asymptotic behavior $\sigma_a \propto E^{-3.5}$. The numerical integration over the entire energy range gives a value of (22.0 $\pm$ 0.7) a.u. for the static dipole polarizability.

TABLE S1. Experimental ionization quantum yields (η), photoabsorption (σ_a), photoionization (σ_i) and neutral-decay (σ_n) cross sections for acetaldehyde. Cross sections are given in units of Mb (10^{-18} cm^2). An overall uncertainty of 10% was determined in the η values, 4% in the σ_a, and 11% in the σ_i and σ_n.

E (eV)	σ_a	σ_i	σ_n	η	E (eV)	σ_a	σ_i	σ_n	η
10.8	31.54				16.1	79.75	72.23	7.42	0.91
10.9	32.30				16.2	79.49	72.61	6.88	0.91
11.0	33.20				16.3	79.23	73.04	6.16	0.92
11.1	34.02				16.4	78.84	73.63	5.25	0.93
11.2	34.79				16.5	78.71	74.29	4.37	0.94
11.3	35.14				16.6	78.39	75.03	3.31	0.96
11.4	36.59				16.7	78.24	75.88	2.30	0.97
11.5	38.59				16.8	78.21	75.99	2.19	0.97
11.6	39.99				16.9	78.39	76.35	2.01	0.97
11.7	41.81				17.0	78.24	77.24	1.11	0.99
11.8	42.81				17.1	78.47	77.77	0.76	0.99
11.9	44.26				17.2	78.77	78.03	0.74	0.99
12.0	46.86				17.3	78.93	78.21	0.74	0.99
12.1	49.17				17.4	79.09	78.28	0.80	0.99
12.2	52.54				17.5	79.31	78.34	0.97	0.99
12.3	53.60				17.6	79.13	78.06	1.09	0.99
12.4	54.46				17.7	78.87	77.70	1.17	0.99
12.5	55.60				17.8	78.72	77.40	1.31	0.98
12.6	55.46				17.9	78.45	77.11	1.38	0.98
12.7	55.88				18.0	78.24	76.77	1.50	0.98
12.8	57.17				18.1	77.93	76.40	1.56	0.98
12.9	57.91				18.2	77.76	76.08	1.61	0.98
13.0	58.91				18.3	77.36	75.59	1.68	0.98
13.1	60.21				18.4	77.17	75.30	1.84	0.98
13.2	61.75				18.5	76.89	74.94	1.91	0.98
13.3	64.70				18.6	76.69	74.68	2.01	0.97
13.4	67.56				18.7	76.48	74.35	2.12	0.97
13.5	69.80	30.94	36.96	0.47	18.8	76.45	74.24	2.17	0.97
13.6	70.43	31.98	37.59	0.47	18.9	76.16	73.90	2.19	0.97
13.7	70.51	34.29	36.34	0.48	19.0	75.99	73.82	2.15	0.97
13.8	69.52	36.40	34.05	0.51	19.1	75.87	73.83	2.03	0.97
13.9	68.22	38.44	31.42	0.54	19.2	75.74	73.86	1.88	0.98
14.0	66.88	39.93	28.86	0.57	19.3	75.79	74.05	1.75	0.98
14.1	65.82	41.11	26.50	0.60	19.4	75.71	74.18	1.52	0.98
14.2	65.71	41.52	24.23	0.63	19.5	75.54	74.26	1.26	0.98
14.3	66.24	44.08	22.24	0.66	19.6	75.42	74.40	1.05	0.99
14.4	67.81	46.85	21.14	0.69	19.7	75.38	74.61	0.79	0.99
14.5	70.68	49.96	20.93	0.70	19.8	75.16	74.66	0.54	0.99
14.6	73.95	52.90	21.28	0.71	19.9	75.32	74.94	0.41	0.99
14.7	76.90	55.21	21.88	0.72	20.0	75.15	74.63	0.54	0.99
14.8	78.82	56.73	22.18	0.72	20.1	75.03	74.47	0.56	0.99
14.9	79.84	57.94	22.00	0.72	20.2	75.06	74.58	0.40	0.99
15.0	80.86	59.33	21.57	0.73	20.3	74.80	74.51	0.24	1.00
15.1	80.68	60.19	20.43	0.75	20.4	74.57	74.33	0.19	1.00
15.2	80.26	61.20	19.04	0.76	20.5	74.36	74.21	0.13	1.00
15.3	79.39	62.16	17.26	0.78	20.6	74.13	73.94	0.14	1.00
15.4	80.00	64.64	15.45	0.81	20.7	73.94	73.72	0.17	1.00
15.5	80.41	66.67	13.75	0.83	20.8	73.54	73.30	0.23	1.00
15.6	80.40	68.28	12.18	0.85	20.9	73.16	72.90	0.21	1.00
15.7	80.23	69.39	10.77	0.87	21.0	72.89	72.69	0.15	1.00
15.8	79.95	70.04	9.91	0.88	21.1	72.44	72.35	0.02	1.00
15.9	80.22	70.79	9.41	0.88	21.2	72.02	72.03	0.06	1.00
16.0	79.95	71.56	8.39	0.90	21.3	71.56	71.48	0.06	1.00

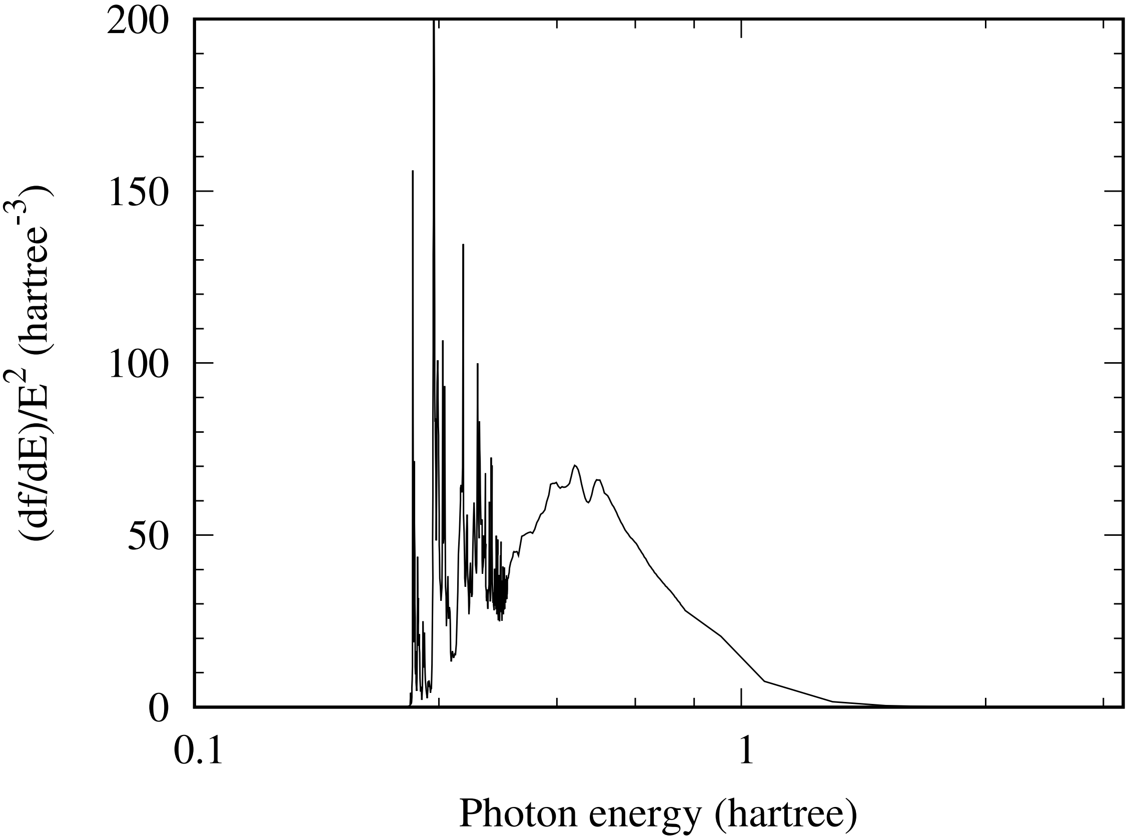

Figure S1. $(df/dE)/E^2$ in function of photon energy for acetaldehyde. Below 0.4 a.u. (10.8 eV) the data were obtained using the σ_a of Limão-Vieira *et al.* [Chem. Phys. Lett. **376**, 737 (2003)]; above 0.8 a.u. (21.5 eV) the data were extrapolated using the asymptotic behavior $\sigma_a \propto E^{-3.5}$. The numerical integration over the entire energy range (sum-rule) gives a value of $S(-2) = (32.2 \pm 0.5)$ a.u. for the static dipole polarizability.
